# Thyroid-Stimulating Hormone Increases HNF-4α Phosphorylation via cAMP/PKA Pathway in the Liver

**DOI:** 10.1038/srep13409

**Published:** 2015-08-25

**Authors:** Yongfeng Song, Dongmei Zheng, Meng Zhao, Yejun Qin, Tingting Wang, Wanjia Xing, Ling Gao, Jiajun Zhao

**Affiliations:** 1 Department of Endocrinology and Metabolism, Shandong Provincial Hospital affiliated to Shandong University, Jinan, Shandong, 250021, China; 2 Institute of Endocrinology and metabolism, Shandong Academy of Clinical Medicine, Jinan, Shandong, 250021, China; 3 Department of pathology, Shandong Provincial Hospital affiliated to Shandong University, Jinan, Shandong, 250021, China; 4 Scientific Center, Shandong Provincial Hospital affiliated to Shandong University, Jinan, Shandong, 250021, China

## Abstract

Hepatocyte nuclear factor-4 alpha (HNF-4α) is an orphan nuclear receptor with important roles in hepatic metabolism. Protein phosphorylation plays a functional role in its nuclear localization, DNA binding, and transactivation. Thyroid-stimulating hormone (TSH) is a hormone produced by the anterior pituitary gland, whose direct effect on the metabolic pathway has been observed. Our previous study demonstrated that TSH significantly decreases hepatic nuclear HNF-4α expression. However, whether TSH can influence HNF-4α phosphorylation is unclear. Here, we discovered that TSH can increase HNF-4α phosphorylation and modulate its subcellularlocalization. When HepG2 cells were treated with TSH, the phosphorylation of HNF-4α increased and its nuclear localization was interrupted. Cytoplasmic HNF-4α increased, while nuclear HNF-4α decreased. When the cAMP/PKA pathway was inhibited by the PKA inhibitor H89 and the adenylate cyclase (AC) inhibitor SQ22536, the TSH-mediated phosphorylation of HNF-4α was disrupted. When *Tshr* was silenced in mice, the phosphorylation of HNF-4α decreased, and cytoplasmic HNF-4α decreased while nuclear HNF-4α increased. In conclusion, our study revealed a novel mechanism by which TSH regulated the hepatic HNF-4α subcellular localization, suggesting the possibility that one of the effects of TSH is to reduce the expression of HNF-4α target genes.

Hepatocyte nuclear factor-4α (HNF-4α) is an orphan nuclear receptor (NR) that plays a critical role in hepatocyte differentiation^1^,^2^,^3^, as well as the maintenance of homeostasis of the adult liver, intestines, and pancreatic β cells^4^,^5^,^6^,^7^. Human HNF-4α gene mutations cause maturity onset diabetes of the young 1 (MODY1)^8^,^9^, and the HNF-4α ligands have been extended to include fatty acid metabolites^10^,^11^,^12^.

Hundreds of HNF-4α target genes have been identified in the liver, pancreas, and colon^2^,^4^,^5^. In the liver, some of these targets are genes involved in glucose [PEPCK (*PCK1*), L-pyruvate kinase (L-PK, *PKLR*), glucose-6-phosphatase (G6Pase, *G6PC*)] and xenobiotic and drug metabolism (*e.g., CYP7A1*, C*YP3A4*, and *CYP2D6*). HNF-4α is also known for its regulation of genes involved in lipid transport, especially those encoding apolipoproteins (*APOA1*, *APOA2*, *APOA4*, *APOB*, *APOC2*, *APOC3*, and *APOC4*) that are linked to circulating levels of cholesterol and triglycerides and, hence, to atherosclerosis.

Protein phosphorylation is a common post-translational modification among transcription factors and has been shown to play a functional role in nuclear localization, DNA binding, and transactivation^13^,^14^. HNF-4α binds DNA exclusively as a homodimer and is localized primarily in the nucleus. It contains a highly conserved Ser/Thr residue between the two zinc fingers that is adjacent to a nuclear localization signal (S78 in human HNF4). A recent report showed that phosphorylation of that site in HNF-4α resulted in impaired nuclear localization and DNA binding, which would decrease the transcriptional effects of HNF-4α^15^,^16^. Previous studies have demonstrated that protein kinase A (PKA) phosphorylates HNF-4α at serine (Ser, S) 133 and 134 in the DNA binding domain (DBD) and impairs its DNA binding activity^17^. Song *et al*. found that glucagon and cAMP can increase the phosphorylation of HNF-4α, which loses its ability to bind to the CYP7A1 gene and results in the inhibition of human CYP7A1 gene transcription.

Thyroid-stimulating hormone (TSH) is produced by the anterior pituitary gland, whose main function has been widely considered to be to regulate thyroid hormone (TH) synthesis and release in the thyroid alone^18^. The activation of the TSH receptor (TSHR) by TSH can raise cAMP levels and result in the phosphorylation of some nuclear transcription factors, such as cAMP regulatory element-binding protein (CREB)^19^. Our recent study demonstrated that TSH significantly decreases the expression of hepatic nuclear HNF-4α *in vivo* and *in vitro*^20^. However, it is still not clear whether TSH can influence the phosphorylation of HNF-4α.

In this paper, we demonstrated that TSH can increase the phosphorylation of HNF-4α via the cAMP/PKA pathway, which reduces the nuclear translocation of HNF-4α and attenuates its transcriptional activity in the liver. Our results will contribute to understanding the pathophysiological mechanism for the disruption of glucose and lipid homeostasis under thyroid and pituitary diseases accompanied by an elevated TSH level.

## Results

### TSH downregulates nuclear HNF-4α expression by interfering with its nuclear translocation

**Figure 1:**
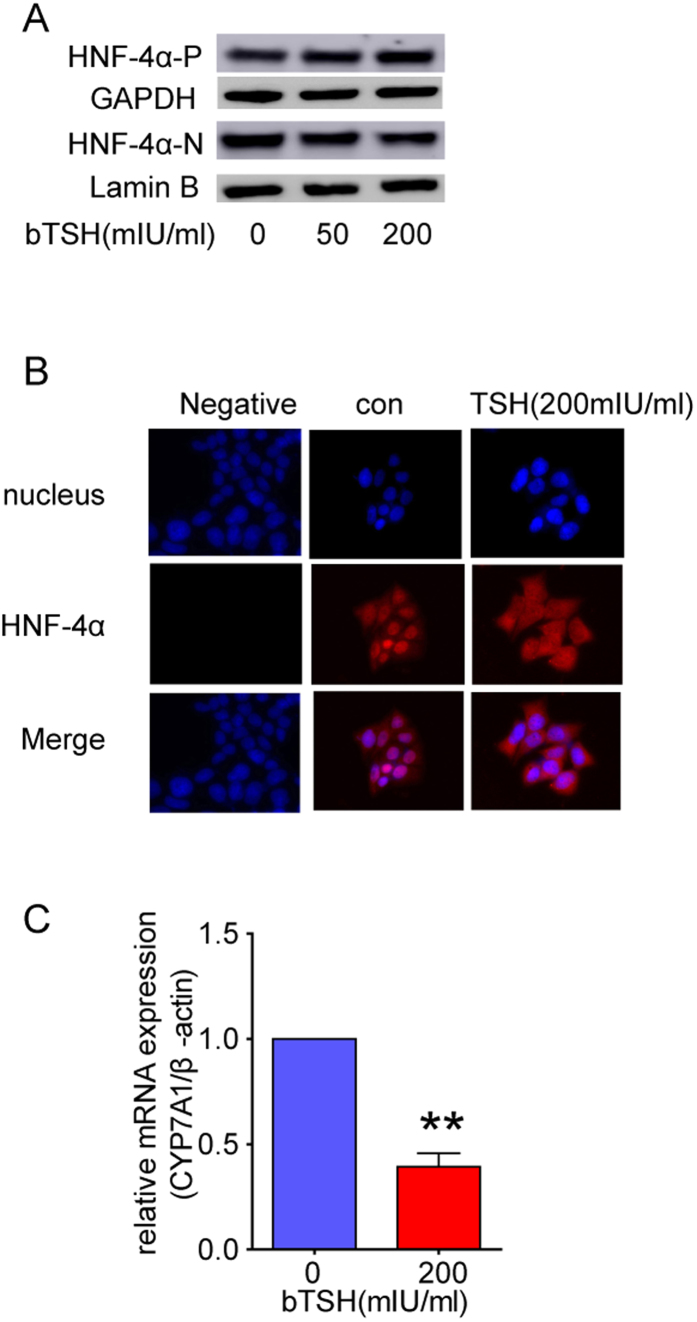
TSH down -regulates nuclear HNF-4α expression by interfering with its nuclear translocation. (**A**)Protein levels of cytoplasmic (-P) and nuclear (-N) HNF-4α were detected in HepG2 cells treated with different dose of bTSH for 48 hours. (**B**) Representative immunofluorescence images were stained using anti-HNF4α antibody (red) and the nuclei were stained by DAPI (blue). Scale bars, 20 μm; magnification: 200×. Images are representative of more than 100 cells. (**C**) Real-time PCR to determine the expression of *Cyp7a1* which is HNF-4α target gene (n = 6). All panels above are representative of 3 independent experiments. *P < 0.05, and **P < 0.01 versus the control (con). The error bars represent the standard deviations. All the gels were run under the same experimental conditions, and key data cropped blots are used here. The full-length gel images are available in the Supplementary Figures.

The HNF-4α transcription factor regulates many liver-specific genes. In the previous study, we demonstrated that the nuclear HNF-4α expression was reduced in HepG2 cells treated with TSH, and its target genes were also regulated^20^. However, besides the change of nuclear HNF-4α protein, higher cytoplasmic levels of HNF-4α were found in the TSH-treated cells (Fig. 1A), suggesting that TSH might interfere with the nuclear localization of HNF-4α. The subcellular localization of HNF-4α was also checked by immuno-fluorescence. In the control HepG2 cells, HNF-4α was quantitatively localized to the nucleus, while in the TSH-treated cells, it was predominantly found in the cytoplasm (Fig. 1B). Meanwhile, as shown in Fig. 1C, the expression of the HNF-4α target genes *Cyp7a1* also decreased, which is consistent with our previous study^20^. These results suggest that TSH interferes with the nuclear localization of HNF-4α, which in turn is responsible for the regulation of HNF-4α target genes.

### The TSH-mediated phosphorylation of HNF-4α via the cAMP/PKA pathway is responsible for its reduced nuclear translocation and transcriptional activity

**Figure 2:**
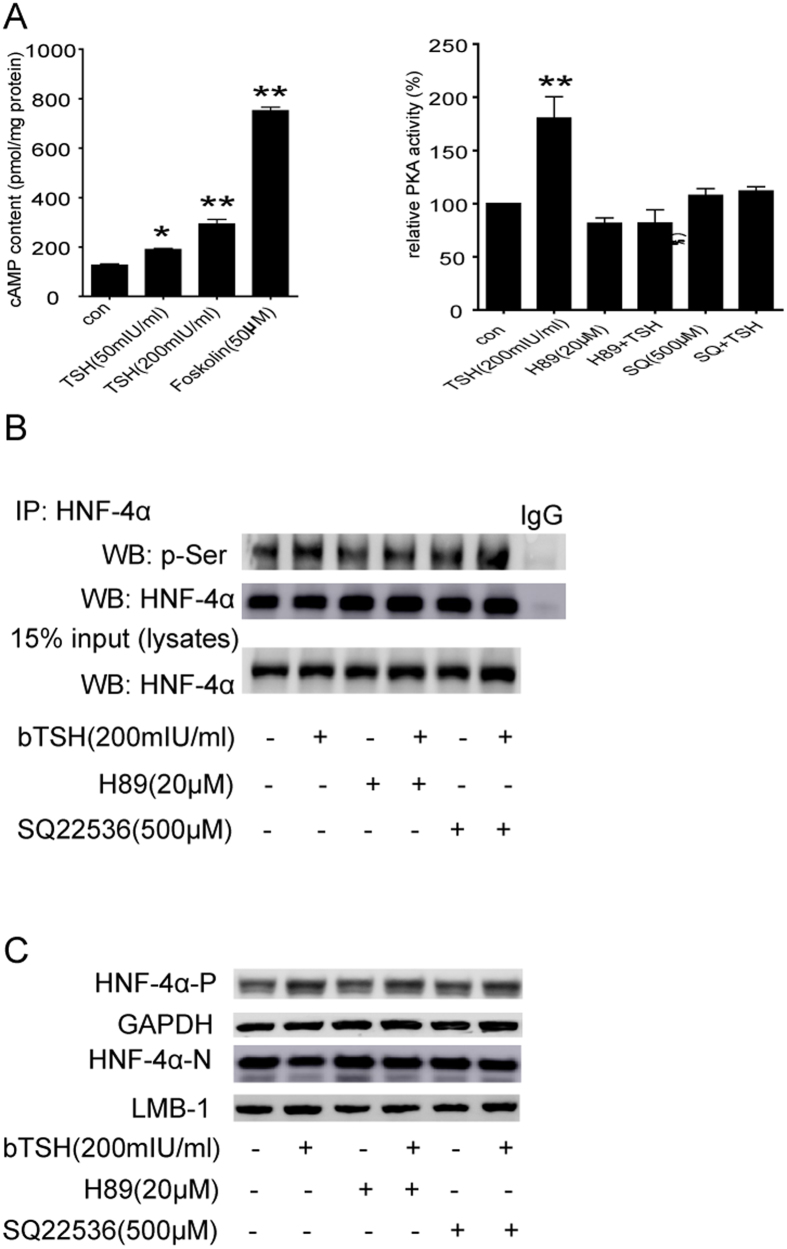
The TSH-mediated phosphorylation of HNF-4α via the cAMP/PKA pathway is responsible for its reduced nuclear translocation and transcriptional activity. (**A**) cAMP content and PKA activity in HepG2 cells were assayed. (**B**) HepG2 cells treated with bTSH for 48 hours in the absence or presence of PKA inhibitor (H89) and AC inhibitor (SQ22536). Cell lysates were purified by immunoprecipitation (IP) with an anti-HNF-4α antibody and were subjected to WB with antibody against phosphorylated-Serine (p-Ser) which represents the phosphorylated levels of HNF-4α. Total lysates were analyzed by WB with anti- HNF-4α antibody as indicated. Normal mouse IgG was used as a negative control. (**C**) The cytoplasmic and nuclear HNF-4α protein levels in HepG2 cells treated with bTSH for 48 hours in the absence or presence of H89 and SQ22536. Representative images from 3 ~ 5 independent experiments are shown. All data are expressed as the mean±standard deviations. **P < 0.01 versus the control (con). The error bars represent the standard deviations. All panels above are representative of 3 independent experiments. All the gels were run under the same experimental conditions, and key data cropped blots are used here. The full-length gel images are available in the Supplementary Figures.

We also demonstrated the possible mechanism that TSH modulates the subcellular localization of HNF-4α. A recent report suggested that phosphorylation of a highly conserved serine (Ser78) in HNF-4α resulted in its impaired nuclear localization^15^. Furthermore, our previous study showed that TSH increases cAMP production via TSHR^21^, and cAMP represses *CYP7A1* via the PKA-mediated phosphorylation of HNF-4α^22^. The system used in this study showed that TSH caused an evident increase in the phosphorylation of HNF-4α; indeed, this phenomenon was disrupted using the PKA inhibitor H89 and the adenylate cyclase (AC) inhibitor SQ22536 in HepG2 cells (Fig. 2A–C). To confirm that the TSH-mediated effect on HNF-4α nuclear localization was due to its phosphorylation, we also observed the subcellular localization of HNF-4α by treating HepG2 cells with H89; the levels of nuclear HNF-4α were partially but not completely reversed by H89 and SQ22536 (Fig. 2D), which suggests that pathways other than the cAMP/PKA pathway may also be involved in the TSH-mediated repression of nuclear HNF-4α, for example, the PI3K/Akt pathway^20^. Together, these results confirm that TSH increases HNF4 phosphorylation through the activation of cAMP/PKA pathways. This is responsible for the reduced nuclear localization and transcription factor activity of HNF-4α.

### The effect of TSH on HNF-4αphosphorylation is dependent on TSHR

**Figure 3:**
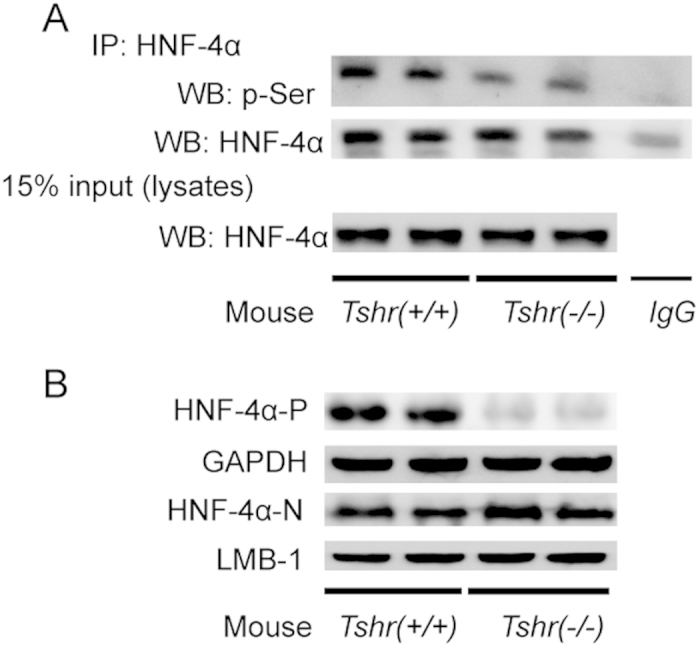
The effect of TSH on HNF-4α phosphorylation is dependent on TSHR. (**A**) Total protein (500 μg) from *Tshr*(*+/+*) and *Tshr*(−/−) mouse liver extracts were purified by IP with the HNF-4α antibody and subjected to WB with the p-Ser antibody. Total lysates were analyzed by WB with antibody against HNF-4α as indicated. Normal mouse IgG was used as a negative control. (**B**) Protein levels of cytoplasmic (-P) and nuclear (-N) HNF-4α were detected in *Tshr*(*+/+*) and *Tshr*(−/−) 8-week old littermate mice. All panels above are representative of 3 independent experiments. All the gels were run under the same experimental conditions, and key data cropped blots are used here. The full-length gel images are available in the Supplementary Figures.

The characteristics of *Tshr*(−/−) mice are described in Table 1 and the experimental procedures section. The serum total T4 (TT4), free T4 (FT4) and TSH levels showed no differences between the wild-type and the *Tshr*-KO littermate mice (all *p* > 0.05).Compared with the littermate wild-type mice, the phosphorylation of HNF-4α decreased in the *Tshr*-KO mice (Fig. 3A). The cytoplasmic HNF-4α decreased, while the nuclear HNF-4α increased (Fig. 3B).

All of these results demonstrate that the effect of TSH on HNF-4α phosphorylation is dependent on TSHR in the liver.

## Discussion

The nuclear receptor HNF-4α is an important regulator of hepatic metabolism^23^. We have demonstrated that TSH significantly decreased the expression of nuclear HNF-4α *in vivo* and *in vitro*^20^. In the present study, our main finding is the novel effect of TSH on HNF-4α phosphorylation via the cAMP/PKA pathway, which regulates the subcellular localization of HNF-4α.

The cAMP/PKA pathway plays an important role in TSH stimulation. PKA has been reported to phosphorylate the A-box of the HNF-4α DNA-binding domain and decrease its DNA-binding activity^17^. Phosphorylation is an important ligand-independent mechanism that regulates essentially every aspect of nuclear receptor (NR) function, just as it does that of other transcription factor families^15^. NRs are phosphorylated in their transactivation domains [the activation function 1 (AF-1) region and the ligand binding domain (LBD)] and the DNA binding domain (DBD)^24^,^25^. Phosphorylation of the DBD has also been shown to impair receptor dimerization and the DNA binding activity of several NRs^24^. Because several NR nuclear localization (NLS) and export signals (NES) are located in the DBD, it is possible that phosphorylation of the DBD will also affect the nuclear localization of NRs. In our study, the nuclear localization of HNF-4α was affected by TSH-mediated phosphorylation.

Interestingly, HNF-4α has been suggested to play numerous roles in many metabolic pathways, such as glucose homeostasis and lipid metabolism [7]. Subclinical hypothyroidism (SCH) is a type of thyroid function abnormality that is defined as increased serum TSH concentrations and normal serum TH levels^26^. Many studies have found that the glucose and lipid metabolism were also disrupted^27^. Our study may partially explain the mechanism by which glucose and lipid disorders occur in SCH patients.

Our study also has some limitations. For example, HNF-4α has been reported to be phosphorylated by multiple kinases at multiple sites. PKA phosphorylates HNF-4α at S133 and 134 in the DBD and impairs its DNA binding activity^17^. AMP-activated protein kinase phosphorylates HNF-4α at S304 in the LBD and impairs its dimerization and DNA binding activity^28^. p38 kinase phosphorylates S158 in the LBD of HNF-4α in response to inflammatory redox, causing an increase in DNA binding and transactivation^28^. Further study is needed to demonstrate the exact phosphorylation site for TSH-mediated regulation of HNF-4α.

In summary, our study demonstrated a novel mechanism in which TSH regulated the hepatic HNF-4α subcellular localization. By regulating HNF-4α localization and thereby its transcription factor activity, TSH is likely to modulate the expression of several hepatocyte genes, which could contribute positively to a pathophysiological mechanism for the disruption of glucose and lipid homeostasis.

## Materials and Methods

### TSHR-knockout mice

The Shandong Provincial Hospital Animal Care and Use Committee approved the procedures for all animal experiments, and the methods were carried out in accordance with the approved guidelines. *Tshr*-knockout (KO) (*Tshr*^tm1Rmar^, genetic background C57BL/6J) heterozygous mice [*Tshr*(+/−)] were obtained from the Jackson Laboratory, USA. Heterozygous mice were intercrossed to produce the homozygous mice[*Tshr*(−/−)] used in this study. The genotyping was determined from tail DNA. Because of the absence of *Tshr* gene expression, serum T4 was undetectable in *Tshr*(−/−)mice. To exclude the effect of thyroid hormones, the mice were placed on a hormone-replacement diet supplemented with 100 ppm desiccated thyroid powder (Sigma).Wild-type [*Tshr*(+/+)]C57BL/6 mice were used as a control. The mice were housed at 23 °C in a 12-hour light-dark cycle (7 A.M. on, 7 P.M. off) and humidity-controlled (60%) environment.At 6–8 weeks of age, littermate male mice were sacrificed between 10:00 P.M. and 2:00 A.M. after fasting for approximately 12 h. Blood samples from the animals were obtained for analyses of serum levels of T_4_, TSH and liver function. In addition, liver tissues from all animals were collected. A partial tissue sample was fixed in freshly prepared 4% paraformaldehyde in phosphate-buffered saline for histological analysis. The remaining tissue was immediately stored in liquid nitrogen until further analysis.

### Cell culture and treatment

The human HepG2 hepatocellular carcinoma cell line and the human L02 liver cell line were obtained from the Type Culture Collection of the Chinese Academy of Sciences, Shanghai, China. The HepG2 cells and the L02 cells were cultured in EMEM medium and RPMI 1640 medium with 10% FBS, respectively. As appropriate for the experiments to be performed, the cells were washed twice with PBS and then starved in serum-free medium overnight before they were treated with bovine TSH (bTSH, Sigma, T8931) for 24 h or 48 h. For some experiments, the PKA inhibitor H89 (20 μM) was added 1 hour before TSH treatment in serum-free medium.

### Total, nuclear and cytoplasmic protein extraction

Liver tissue samples were ground into powder in liquid nitrogen. The tissue powder and cells were lysed in RIPA buffer to obtain total protein according to the manufacturer’s protocol. The nuclear and cytoplasmic proteins were prepared using NE-PER Nuclear and Cytoplasmic Extraction Reagents (Pierce). The protein concentration was measured using the BCA method.

### Western blotting

Equal amounts of protein from different samples were subjected to 10% SDS-PAGE followed by electrotransfer from the gel to polyvinylidene difluoride membranes (Millipore). The membranes were incubated overnight at 4 °C with anti-CYP7A1 (1:200, Santa Cruz) and anti-HNF-4α (1:1000, Invitrogen) antibodies. β-actin protein was evaluated as a loading control (1:10000, Abcam) for cytoplasmic protein and Lamin B1 protein as a loading control (1:2000, CST) for nuclear protein. Immune complexes were detected using the Enhanced Chemiluminescence Plus Detection System (Amersham).

### Determination of cAMP levels

Cells were incubated in serum-free medium without antibiotics containing 1mM IBMX (3-isobutyl-1-methylxanthie, a cAMP phosphodiesterase inhibitor, Biomol) for 30 minutes, and then treated with 50 mIU/ml bTSH, 200 mIU/ml bTSH or 50 μM forskolin (a cAMP agonist, Sigma) for 1 hour. Intracellular cAMP levels were determined using a commercially available cAMP Direct Immunoassay Kit (BioVision) according to the manufacturer’s instructions and were normalized against total cellular protein concentration.

### Assay of PKA activity

PKA activity was assessed using a kit (Genmed, Shanghai, China) according to the manufacturer’s instructions. In brief, 5 μg protein was added to a buffer containing substrate and then measured at an absorbance of 340 nm at 0 min and 5 min. The difference between the absorbance at the fifth minute and the immediate absorbance represented the activity.

### HNF-4α phosphorylation assay

HNF-4α phosphorylation was assayed according to the method previously described^22^. The tissue powder and cells were lysed in RIPA buffer containing 1% PMSF and 1% phosphatase inhibitor cocktail. The protein concentration was adjusted to 1 μg/μl. The anti-HNF-4α antibody (mouse monoclonal antibody, Invitrogen) was covalently crosslinked onto Protein A/G resin using a Crosslink Immunoprecipitation Kit (Pierce, USA).A total of 500 μg of protein was incubated overnight at 4 °C with the crosslinked antibody. The HNF-4α protein was eluted from the resin without antibody contamination and then subjected to SDS-PAGE on a 10% gel and transferred to a nitrocellulose membrane (Millipore). Phosphor-HNF-4α and HNF-4α proteins were detected using anti-phosphoprotein (Invitrogen) and anti-HNF-4α (Invitrogen) antibodies, respectively.

### Immunofluorescence (IF)

Cells attached to cover slips were washed with PBS twice and fixed in 4% paraformaldehyde. The cells were blocked with serum that was homologous to the secondary antibody, followed by overnight incubation with the primary antibody (mouse anti-HNF-4α). The sections were subsequently incubated with a FITC-conjugated secondary antibody and mounted with DAPI. The analysis and photo-documentation were performed using a fluorescence microscope (Zeiss, Germany).

### Statistical analysis

The data were analyzed using SPSS 17.0 and are expressed as the mean ± standard deviationor number. Differences among the groups were compared using an unpaired Student’s *t*-test for two-group comparisons or a one-way analysis of variance (ANOVA) (Dunnett’s *t* or LSD test) for multiple comparisons. All of the calculated *P* values are two-sided. Differences were considered significant at *P* < 0.05.

## Additional Information

**How to cite this article**: Song, Y. *et al*. Thyroid-Stimulating Hormone Increases HNF-4α Phosphorylation via cAMP/PKA Pathway in the Liver. *Sci. Rep*. **5**, 13409; doi: 10.1038/srep13409 (2015).

## Supplementary Material

Supplementary Information

## Figures and Tables

**Table 1:** General characteristic of wild-type mice and *Tshr*(−/−) (TH-supplemented) mice (mean ± standard deviations).

	*Tshr*(+/+)	*Tshr*(−/−)
N	14	12
BW (g)	27.58 ± 3.74	23.25 ± 3.29*
FT_4_ (pmol/l)	1.92 ± 0.35	1.87 ± 0.55
TT_4_ (μg/dl)	4.43 ± 1.35	4.74 ± 1.12
TSH (pg/ml)	521.78 ± 301.21	479.46 ± 240.42

N: number; BW: body weight; FT_4_: free thyroxine; TT_4_: total thyroxine; TG: triglyceride; *P < .05 versus *Tshr*(+/+).
