# Supplementary material for: Thyroid-Stimulating Hormone Increases HNF-4α Phosphorylation via cAMP/PKA Pathway in the Liver
Source: Sci Rep. 2015 Aug 25;5:13409. doi: 10.1038/srep13409 (PMC4548215; doi:10.1038/srep13409)
Supplement: Supplementary Information [file srep13409-s1.doc]

**Thyroid-Stimulating Hormone Increases HNF-4α Phosphorylation**

**via cAMP/PKA pathway in the liver**

Yongfeng Song^1,2*^, Dongmei Zheng^1,2*^, Meng Zhao^1,2*^, Yejun Qin^3*^, Tingting Wang^1,2^, Wanjia Xing^1,2^, Ling Gao^2,4*^, and Jiajun Zhao ^1,2*^

1 Department of Endocrinology and Metabolism, Shandong Provincial Hospital affiliated to Shandong University, Jinan, Shandong, 250021, China;

2 Institute of Endocrinology and metabolism, Shandong Academy of Clinical Medicine, Jinan, Shandong, 250021, China;

3 Department of pathology, Shandong Provincial Hospital affiliated to Shandong University, Jinan, Shandong, 250021, China;

4 Scientific Center, Shandong Provincial Hospital affiliated to Shandong University, Jinan, Shandong, 250021, China;

* These authors contributed equally to this study

^🖂^ Corresponding authors: Dr. Ling Gao (e-mail: gaoling1@medmail.com.cn) and Dr. Jiajun Zhao (e-mail: jjzhao@ medmail.com.cn)

**Correspondence:** Dr. Jiajun Zhao. Shandong Provincial Hospital affiliated to Shandong University; Institute of Endocrinology, Shandong Academy of Clinical Medicine, 324, Jing 5 Rd., Jinan, Shandong, 250021, China. Tel: +86-13708932909 Fax: +86-531-87068707

Figure 1A

Control bTSH(50) bTSH (200) Control bTSH(50) bTSH (200)

Cytoplasmic HNF-4α


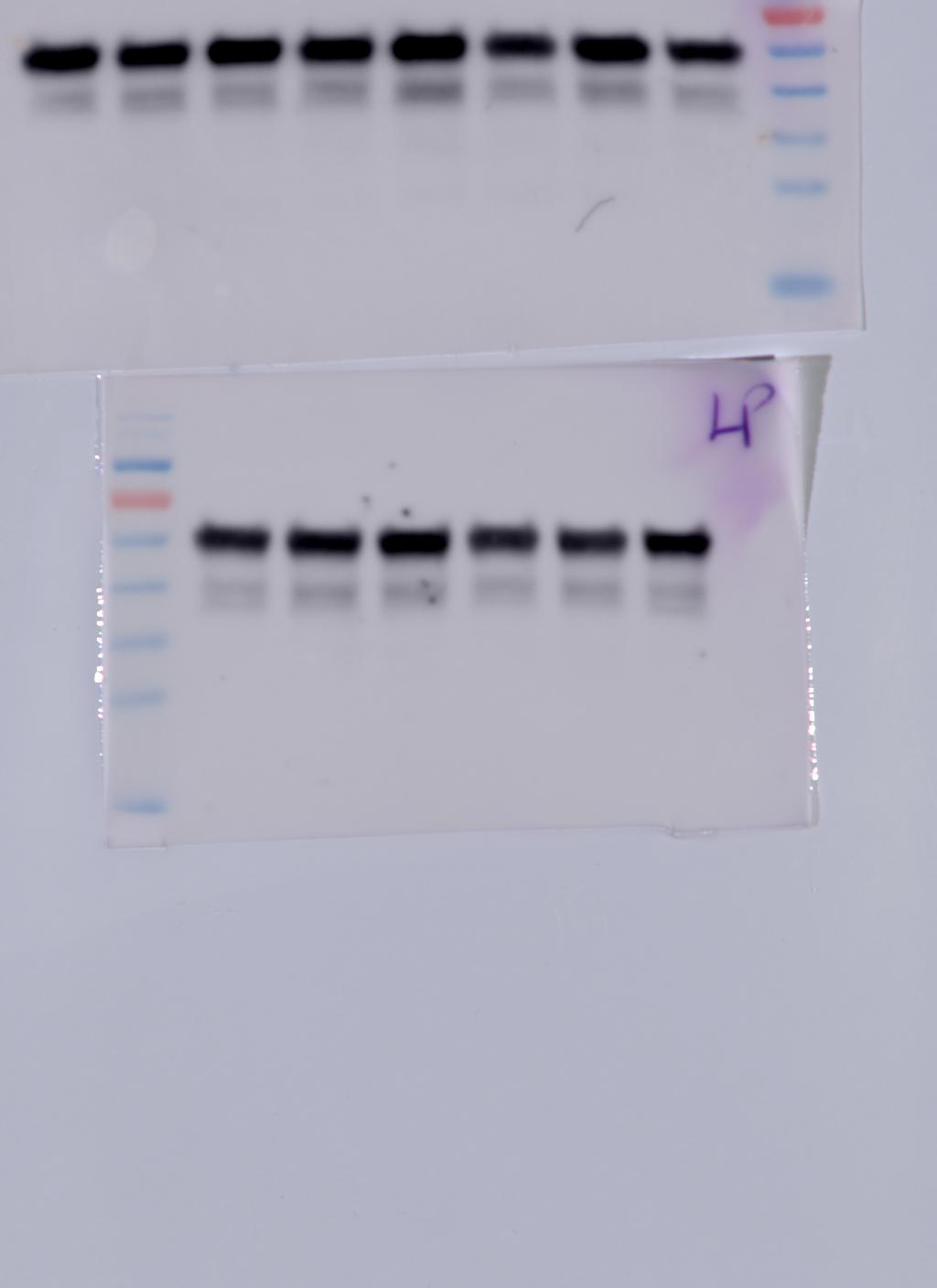


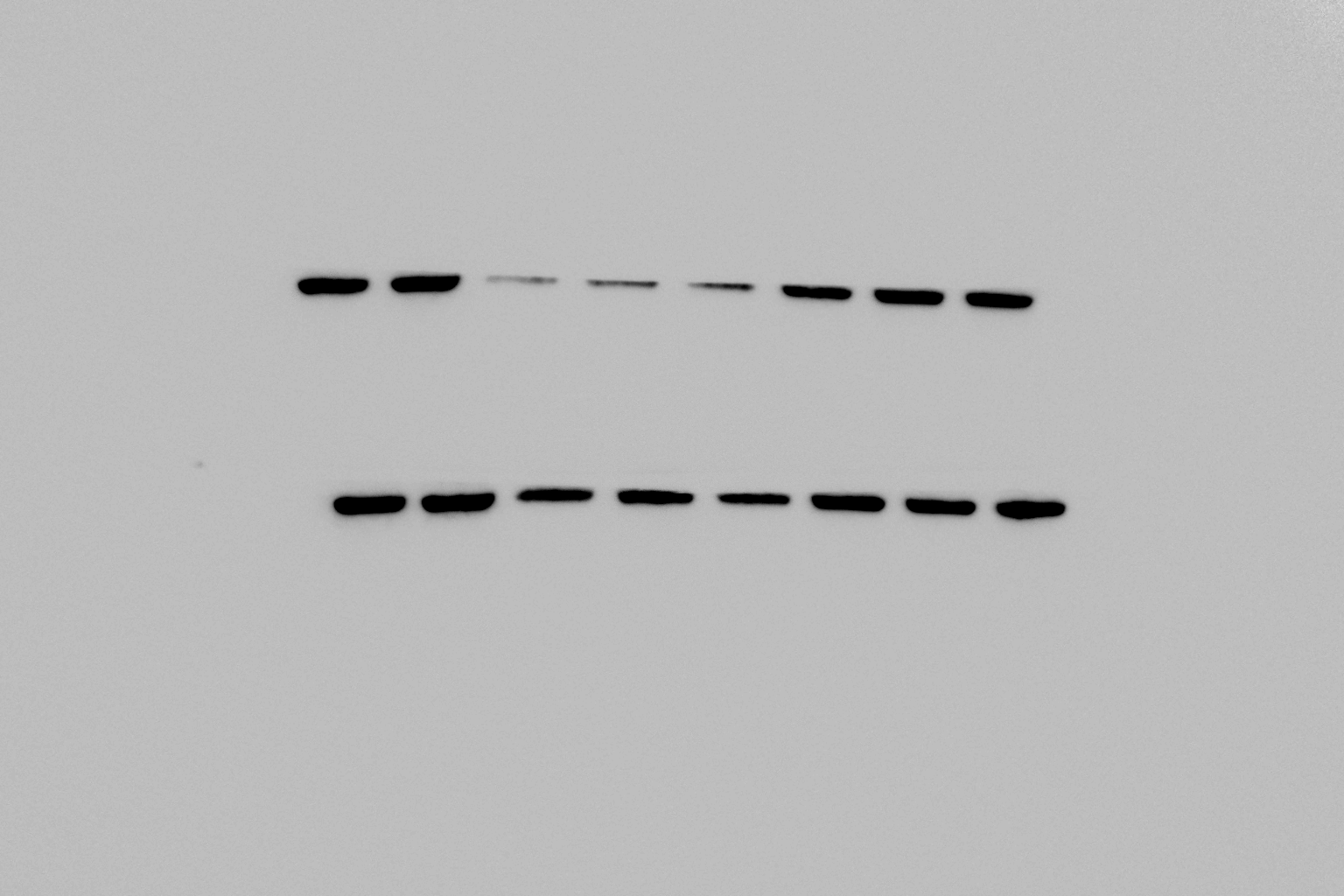


Control bTSH(50) bTSH (200) Control bTSH(50) bTSH (200)

GAPDH


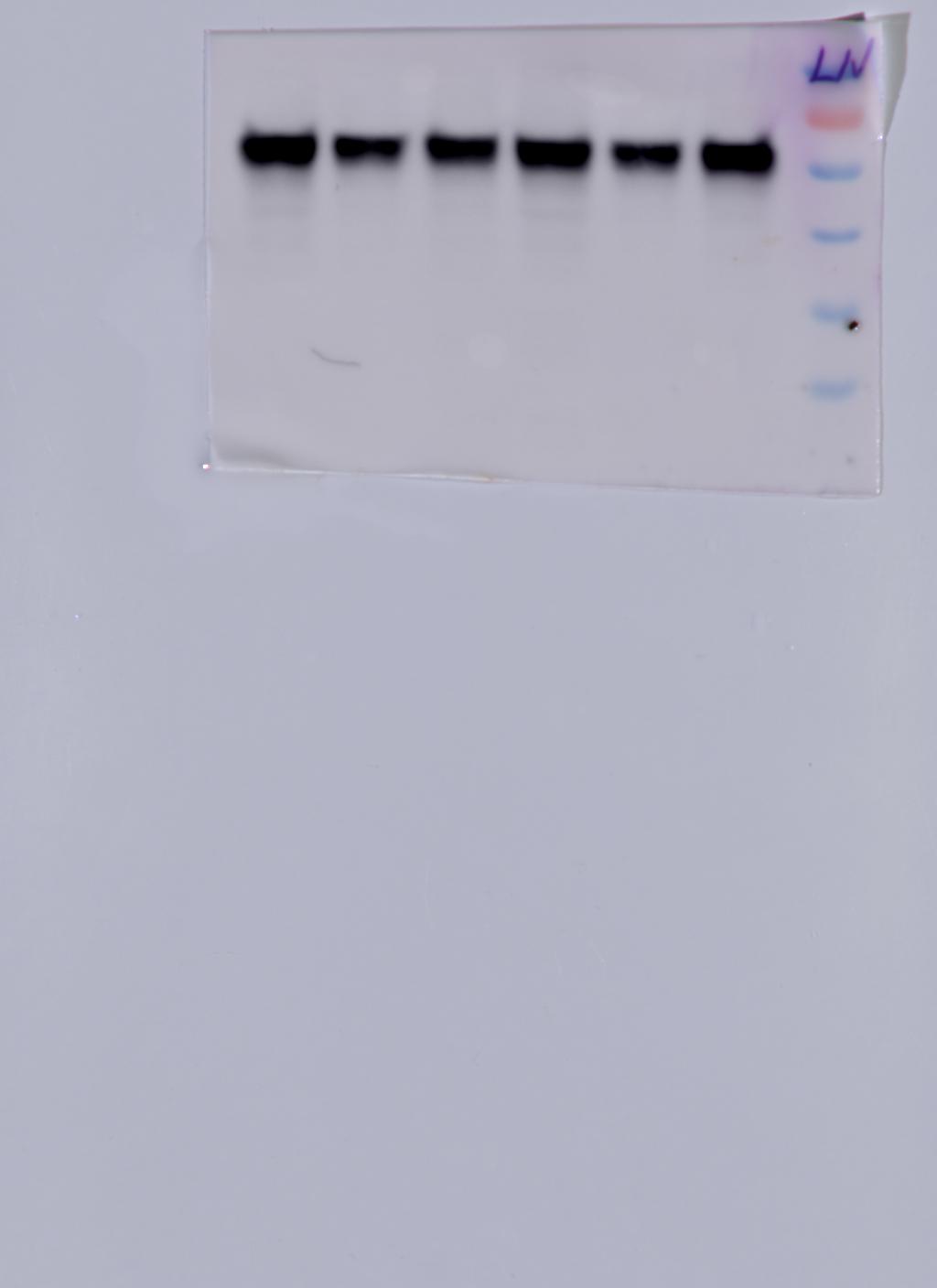


nuclear HNF-4α

bTSH (200) Control bTSH(50) bTSH (200) Control bTSH(200)


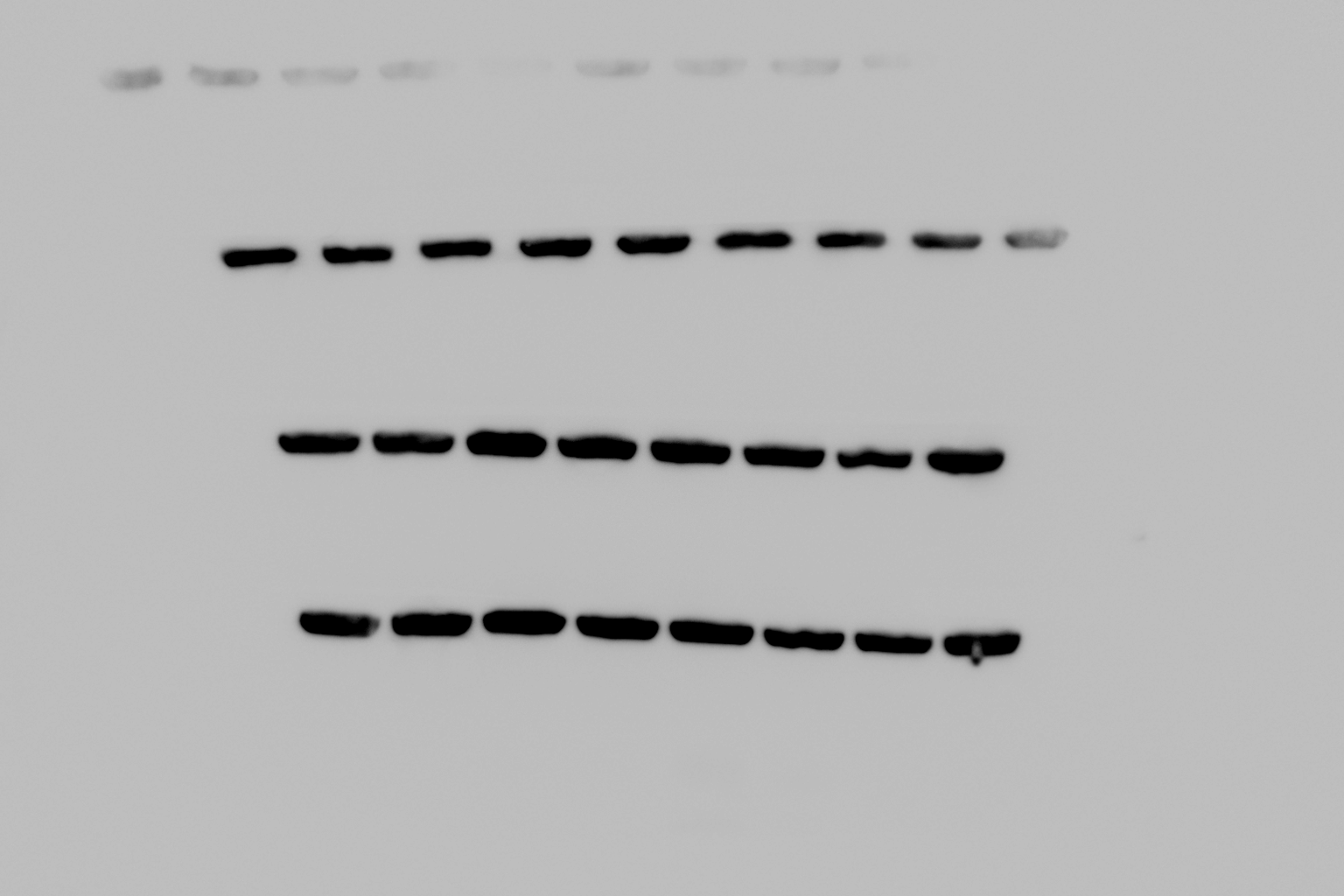


bTSH (200) Control bTSH(50) bTSH (200) Control bTSH(200)

Lamin B 1

Figure 2B


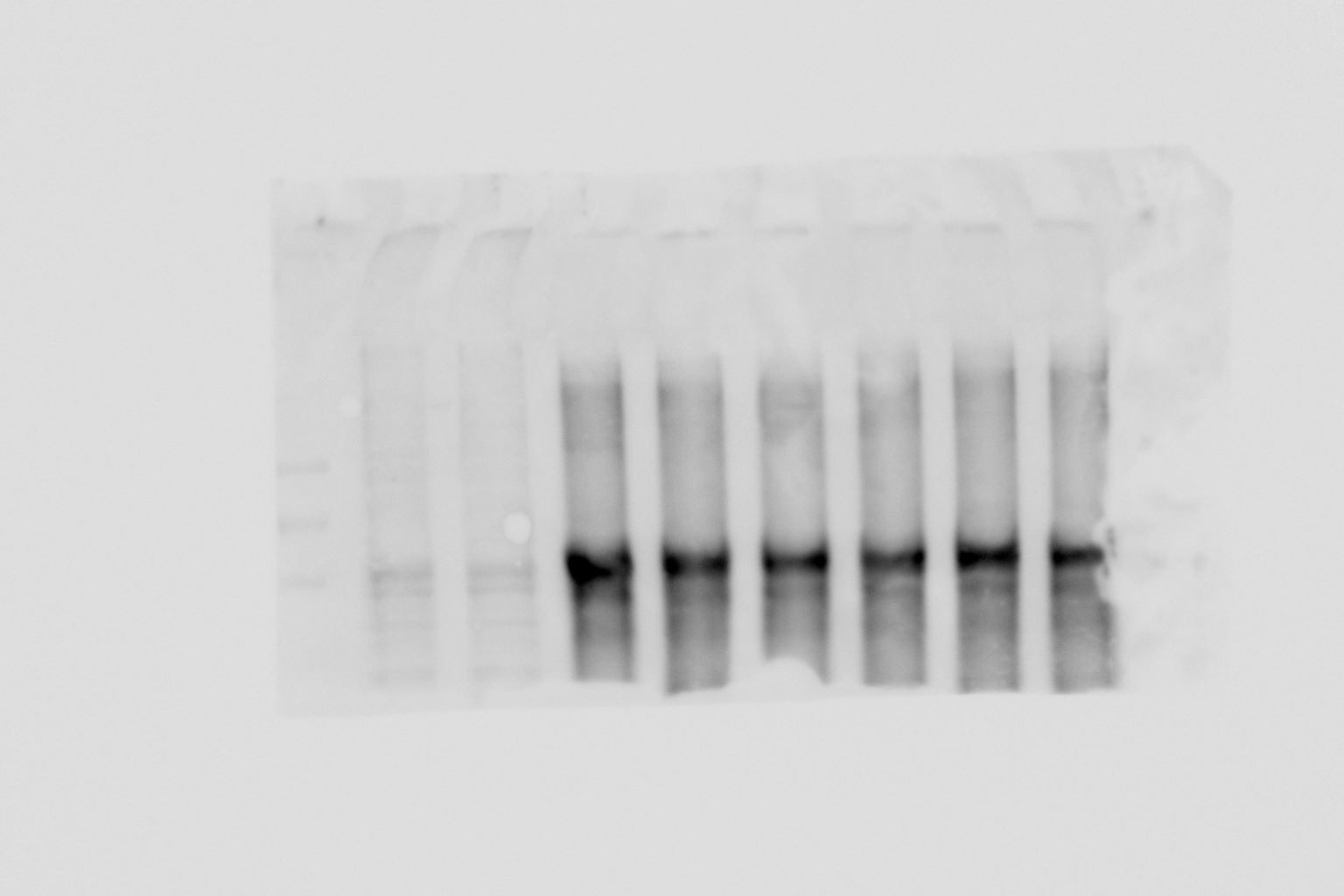


control bTSH(200) H89 H89+TSH SQ22536 SQ+TSH IgG

Phosphor-HNF-4α


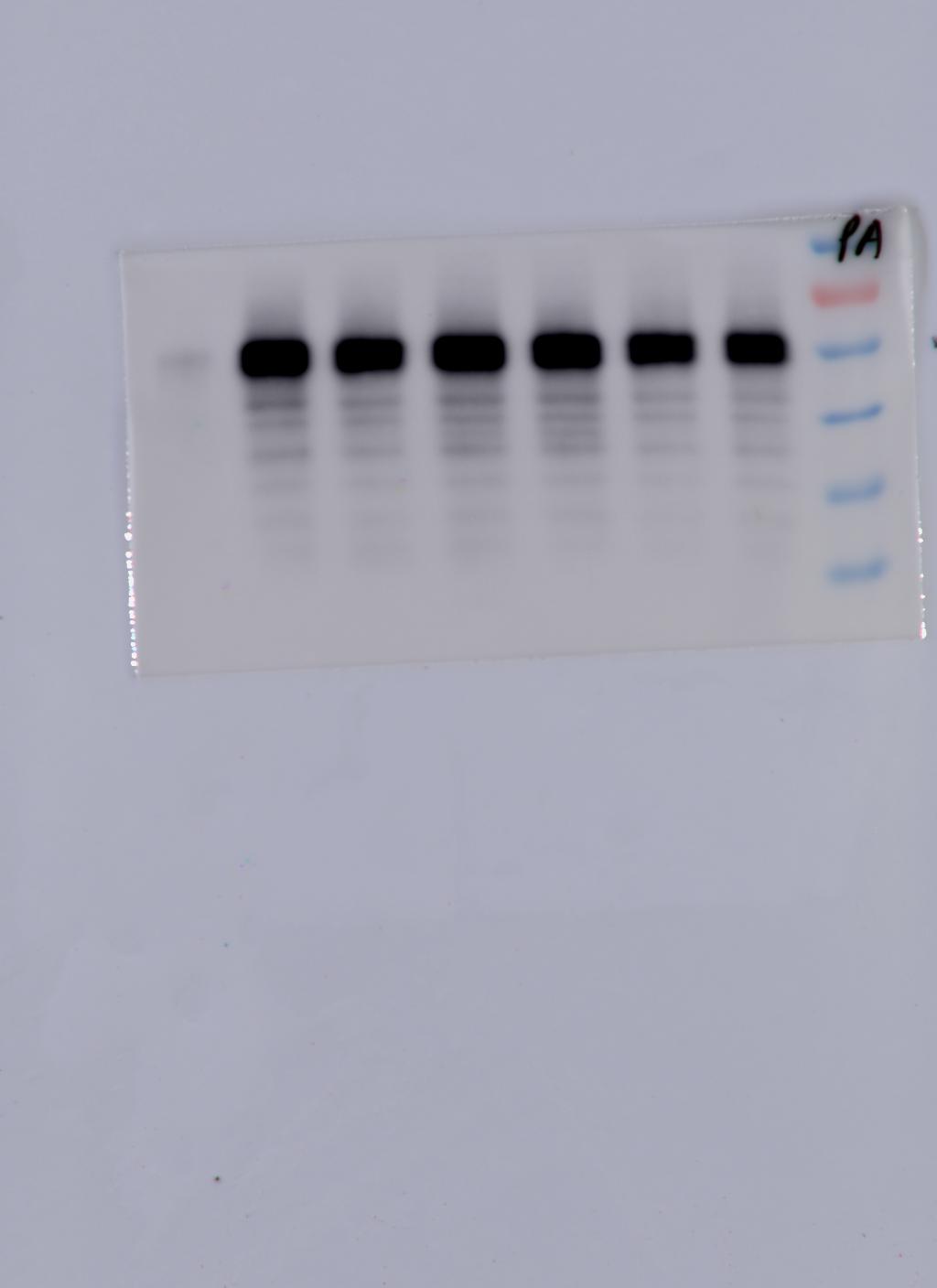


control bTSH(200) H89 H89+TSH SQ22536 SQ+TSH IgG

HNF-4α


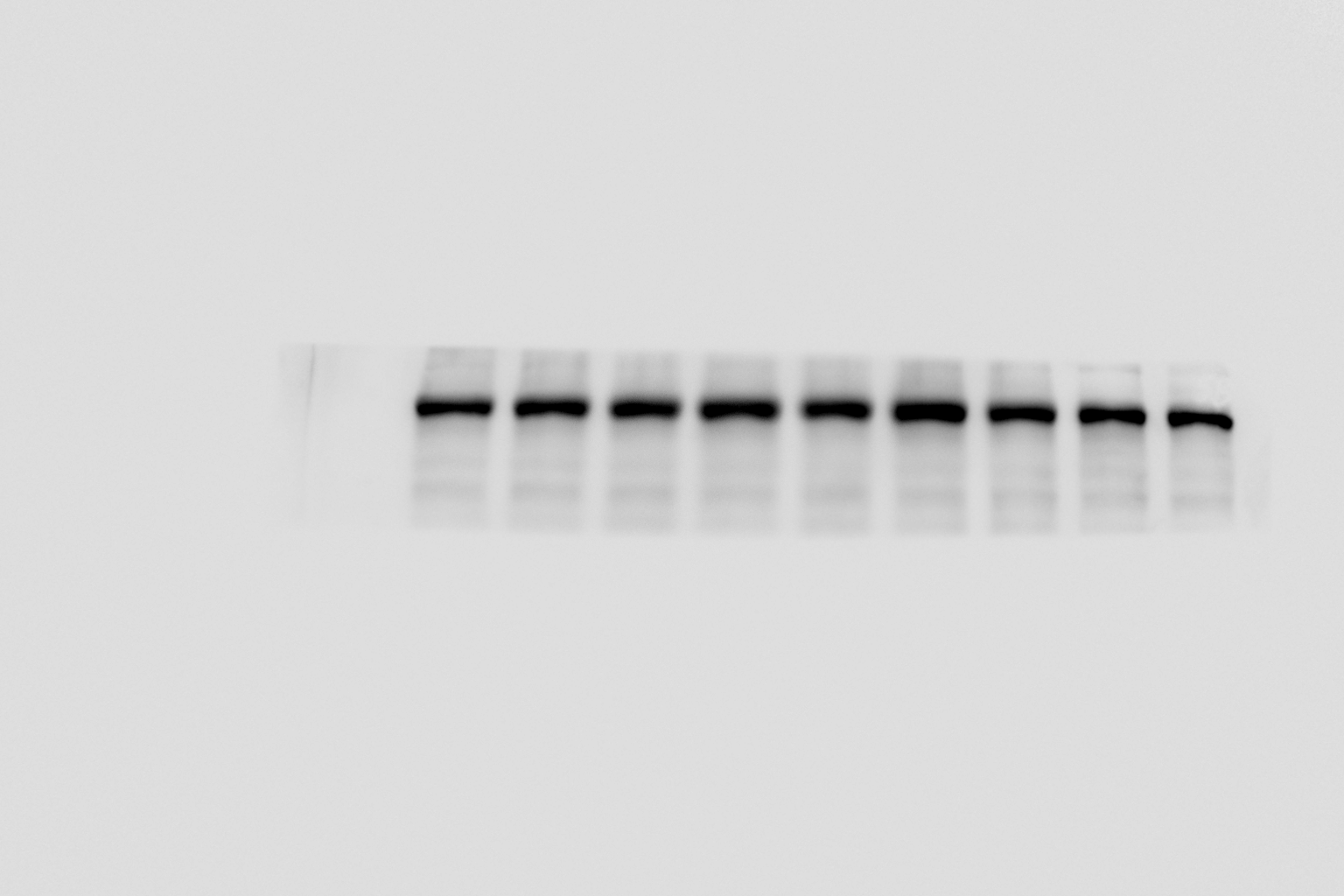


15% input Control bTSH H89 H89+TSH SQ SQ+TSH con bTSH con

HNF-4α

Figure 2C


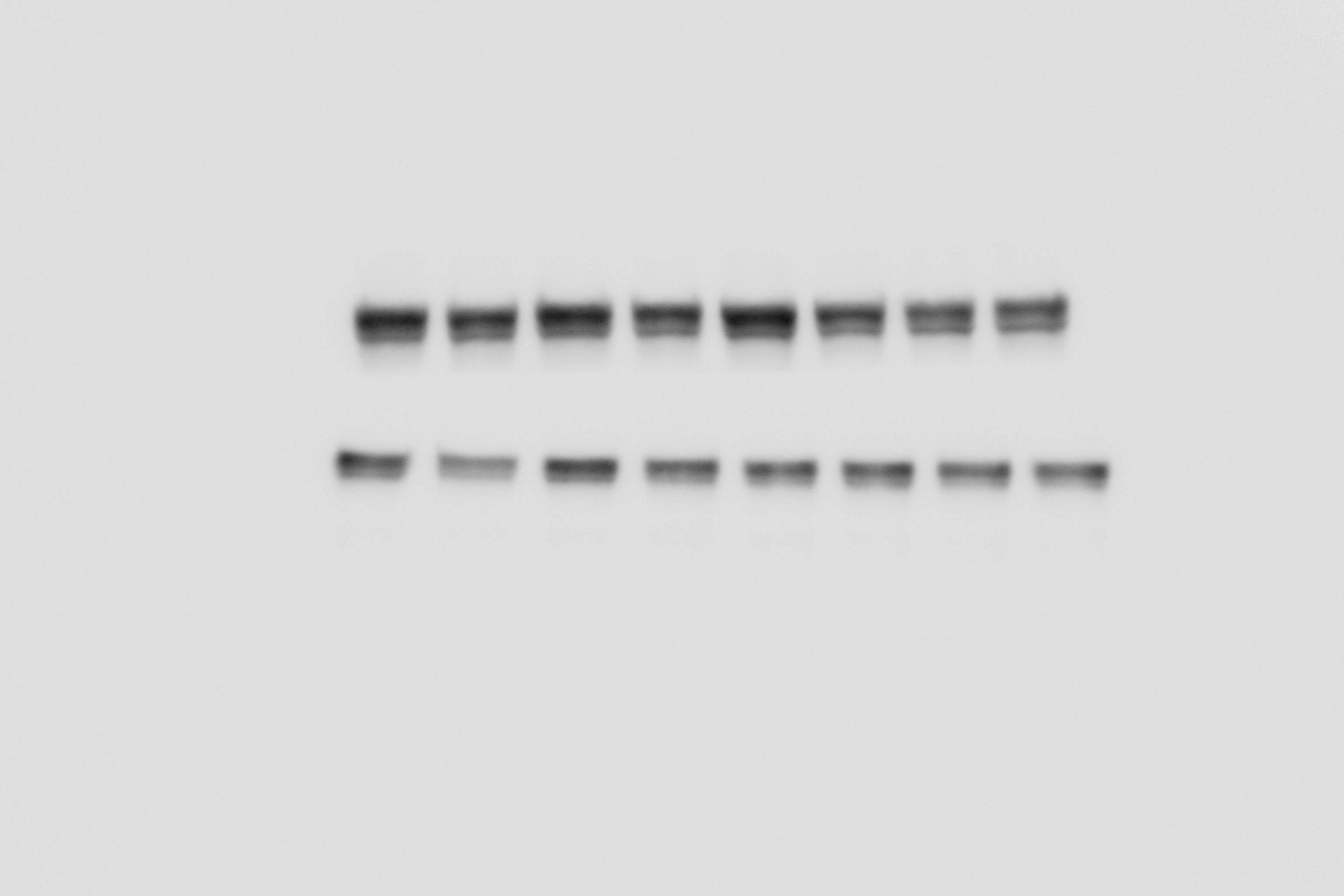


Control bTSH(200) H89 H89+TSH SQ22536 SQ+TSH

Control bTSH(200) H89 H89+TSH SQ22536 SQ+TSH

Cytoplasmic HNF-4α


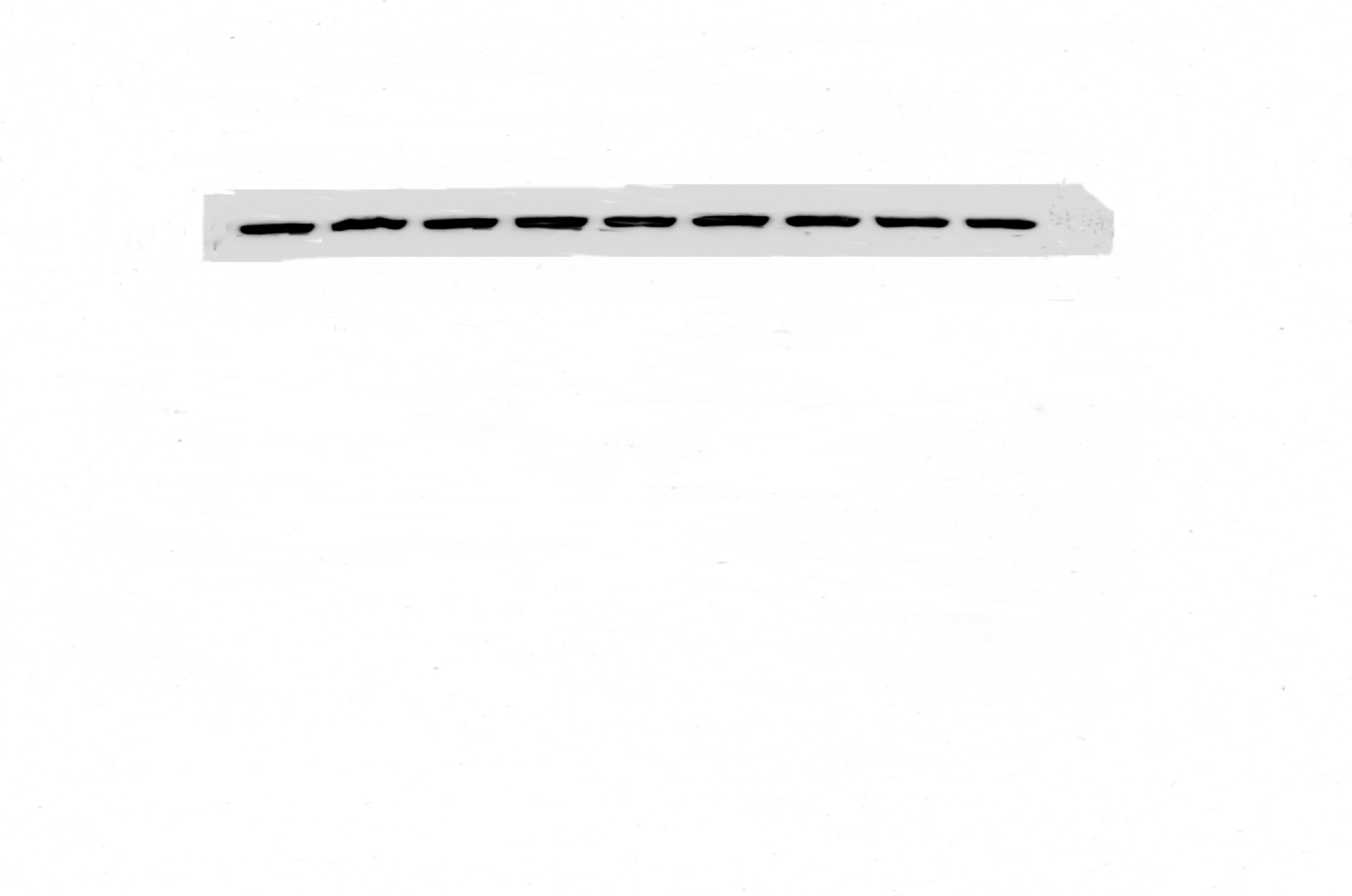


Control bTSH(200) H89 H89+TSH SQ22536 SQ+TSH

GAPDH


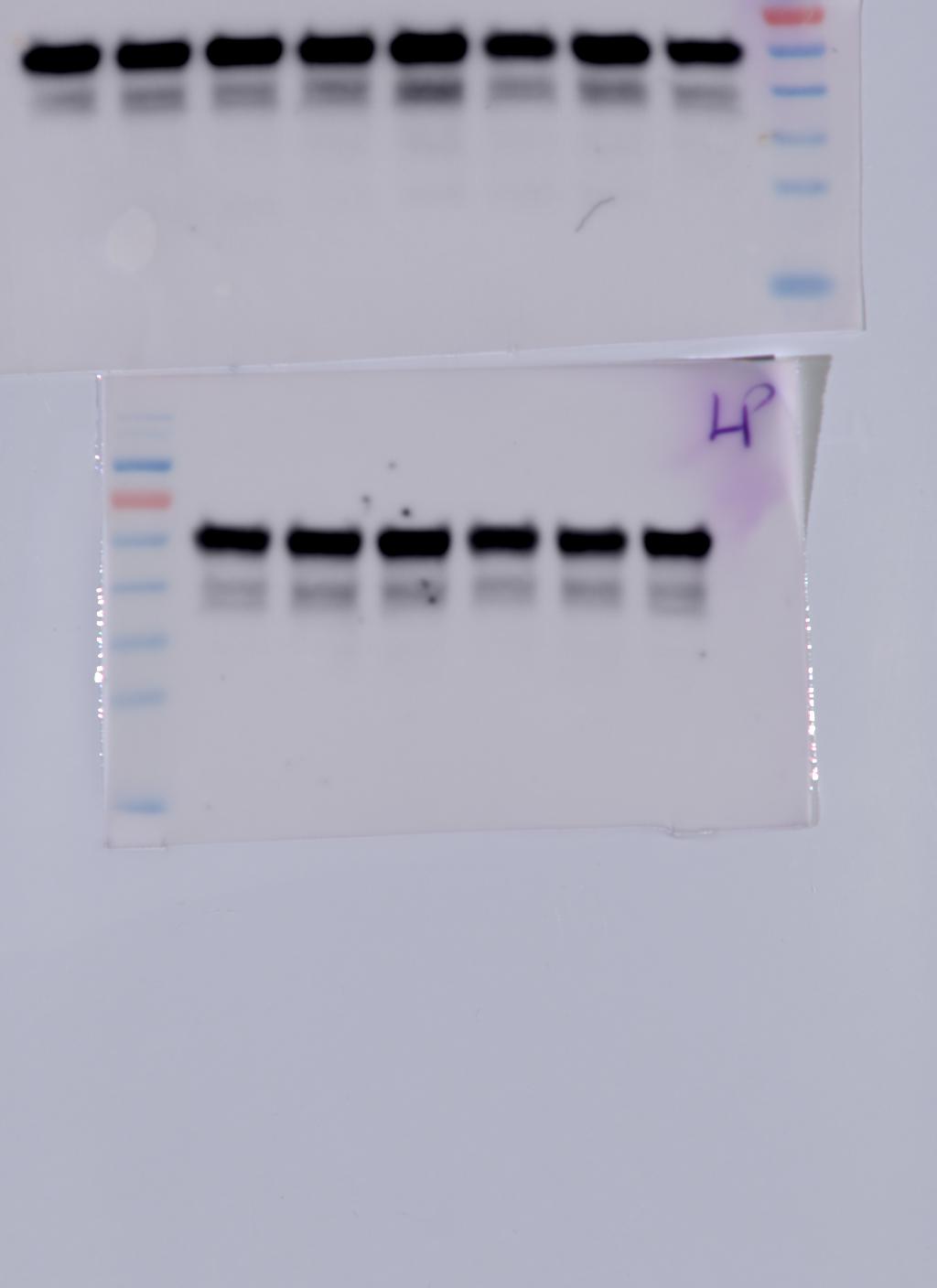


Nuclear HNF-4α


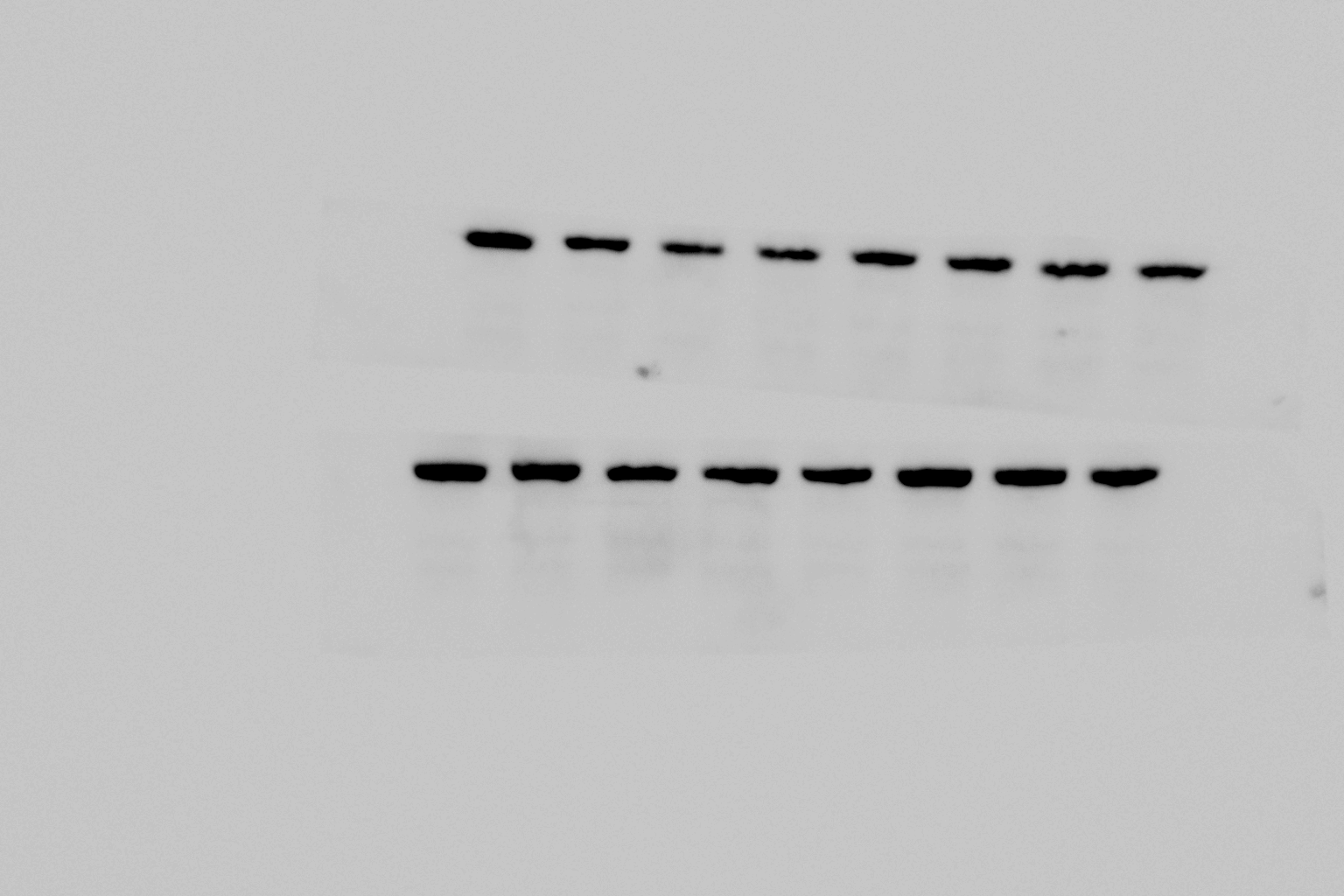


Control bTSH(200) H89 H89+TSH SQ22536 SQ+TSH

Lamin B 1

Figure 3A


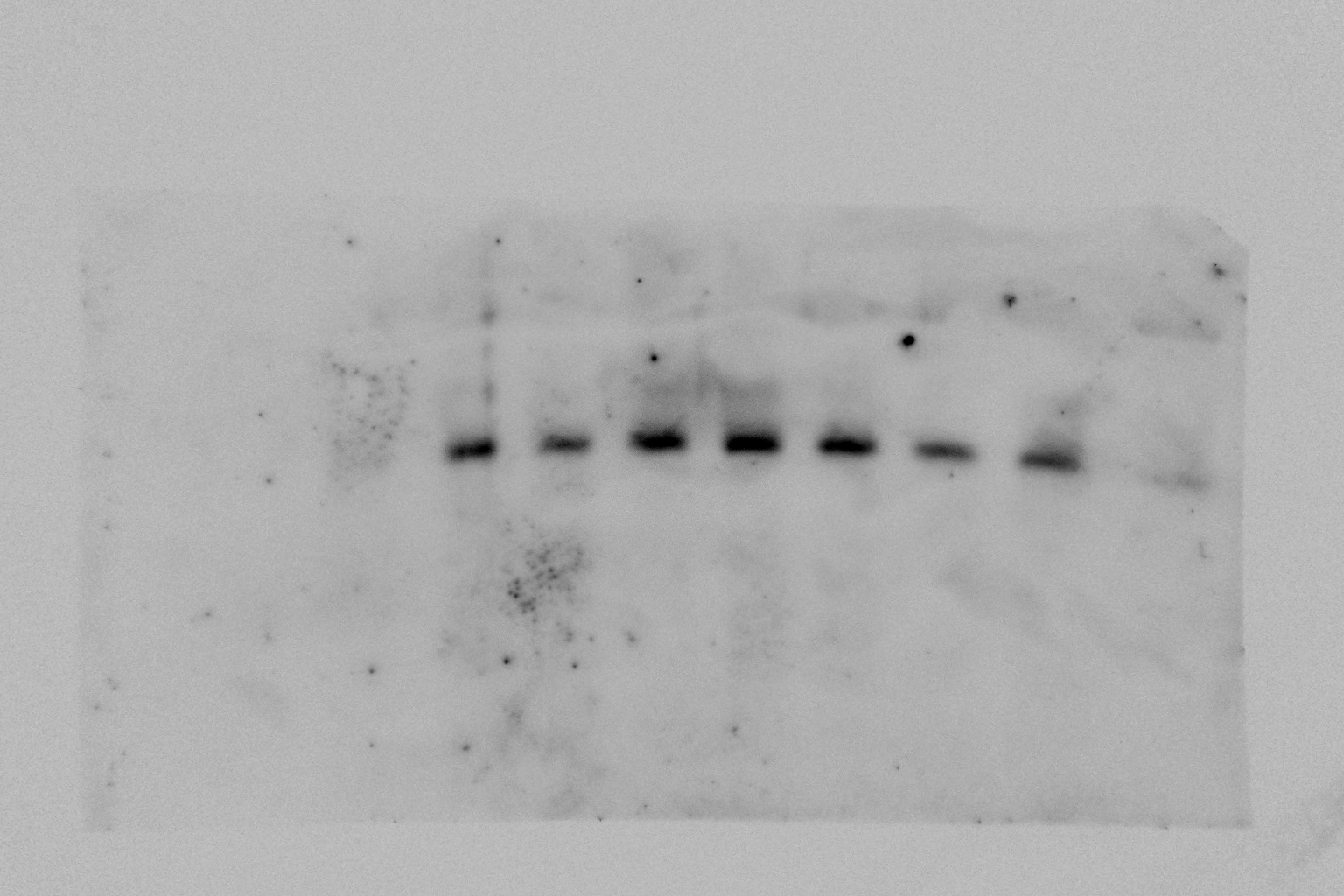


Tshr(+/+)

Tshr(-/-)

Tshr(-/-)

IgG

IgG

Phosphor-HNF4α


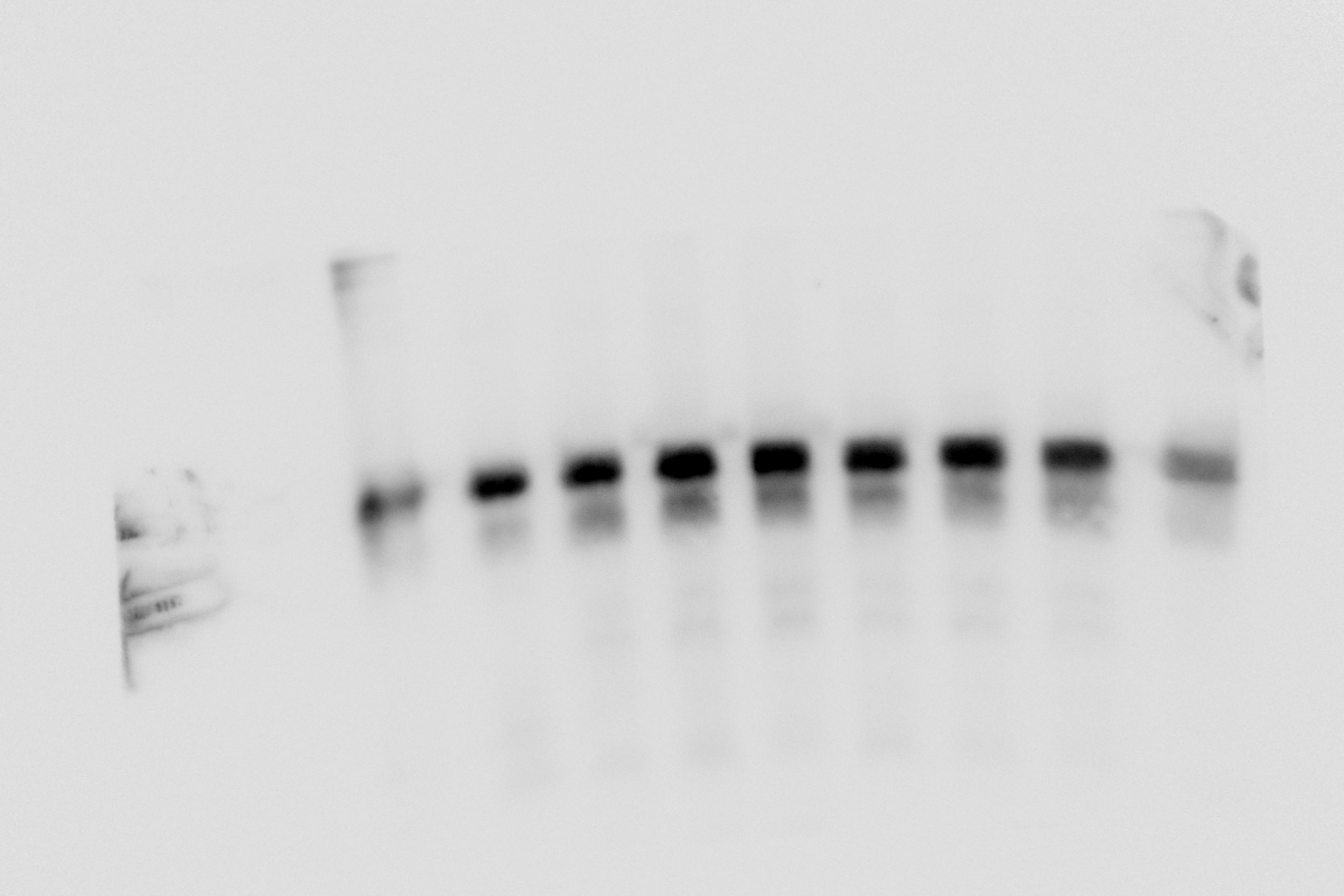


Tshr(+/+)

Tshr(-/-)

Tshr(-/-)

IgG

IgG

HNF4α


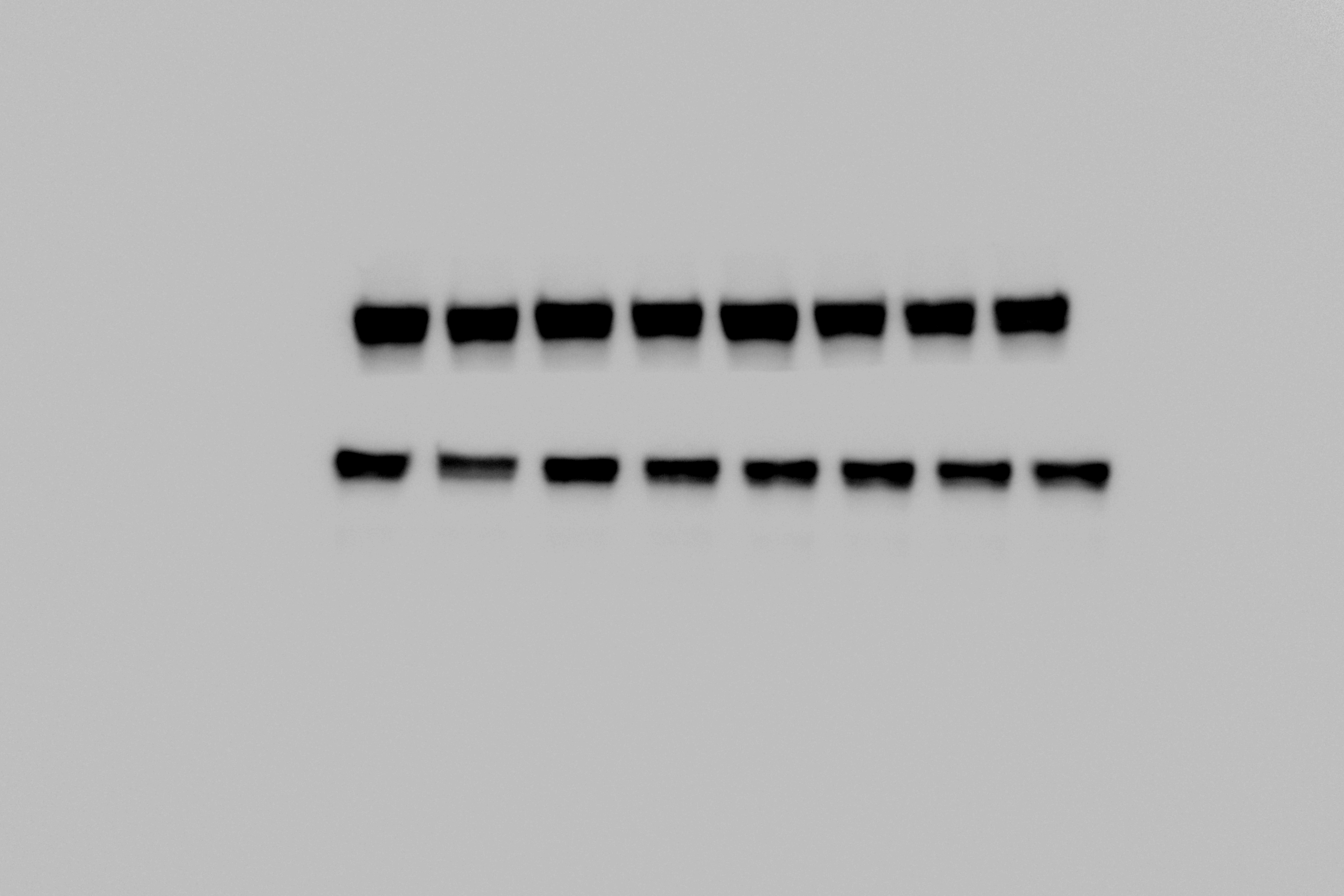


Tshr(+/+)

Tshr(-/-)

Tshr(+/+)

Tshr(-/-)

HNF4α

15% input

Figure 3B


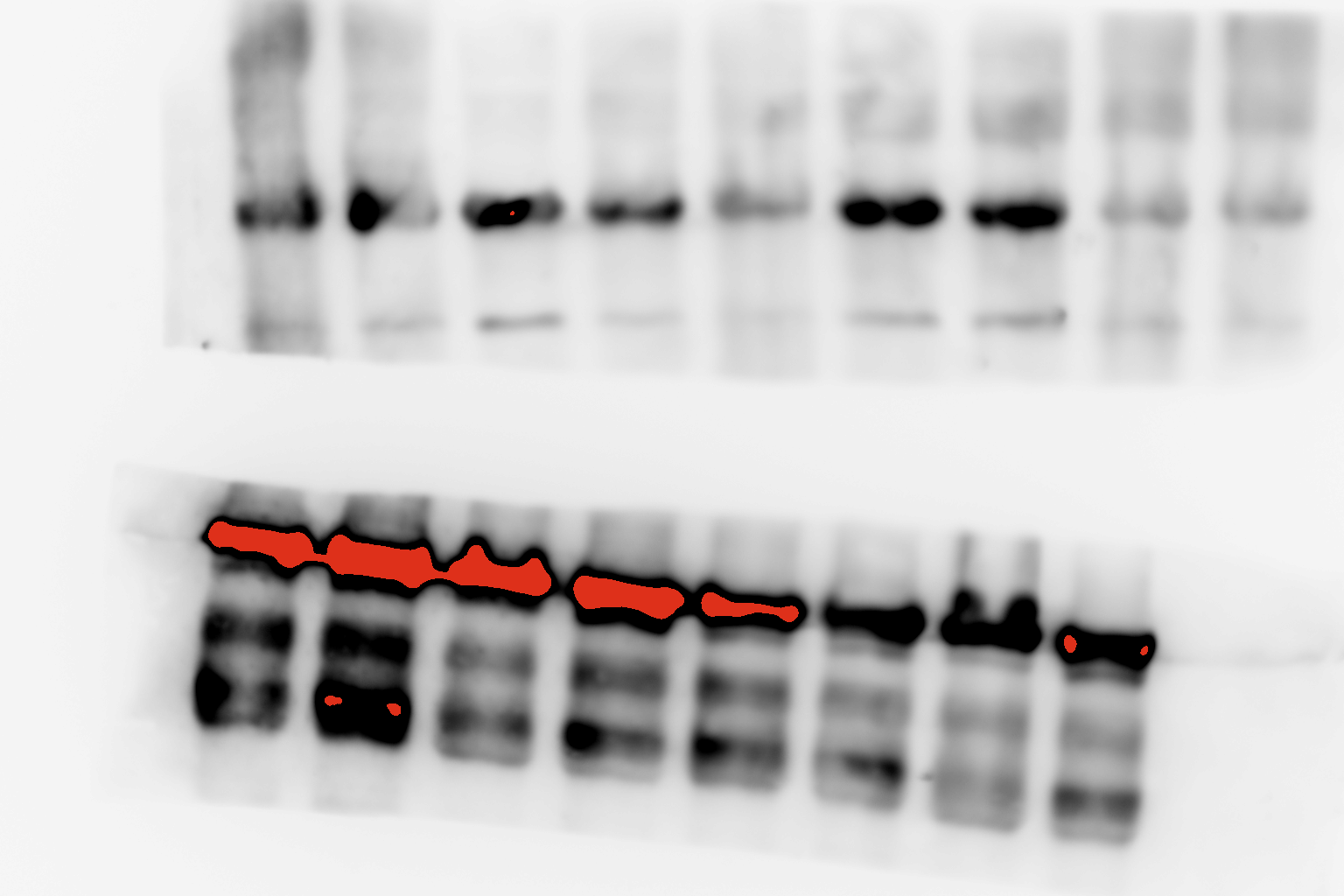


Tshr(+/+)

Tshr(-/-)

Tshr(+/+)

Tshr(-/-)

Cytoplasmic HNF4α


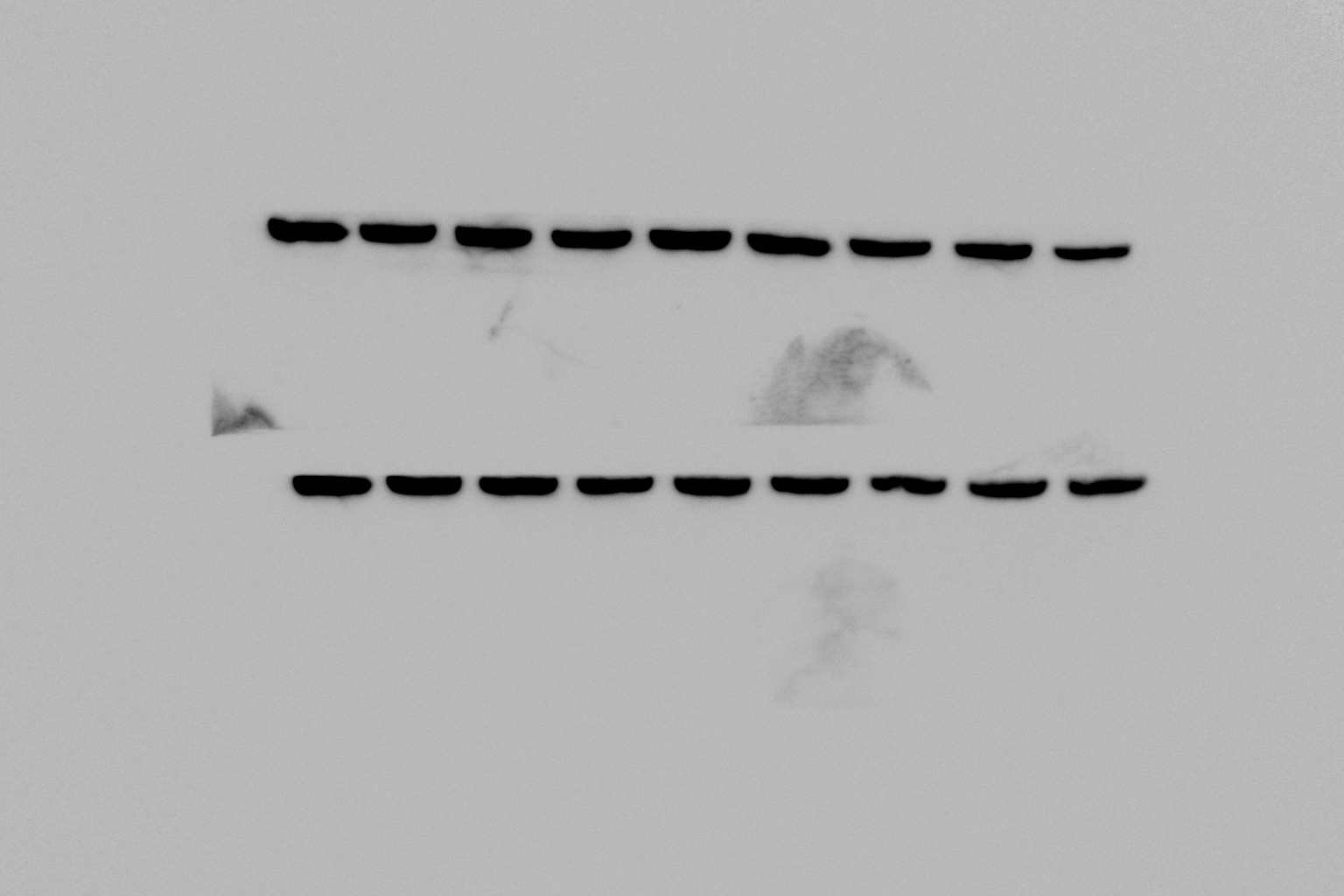


Tshr(+/+)

Tshr(-/-)

Tshr(+/+)

Tshr(-/-)

GAPDH


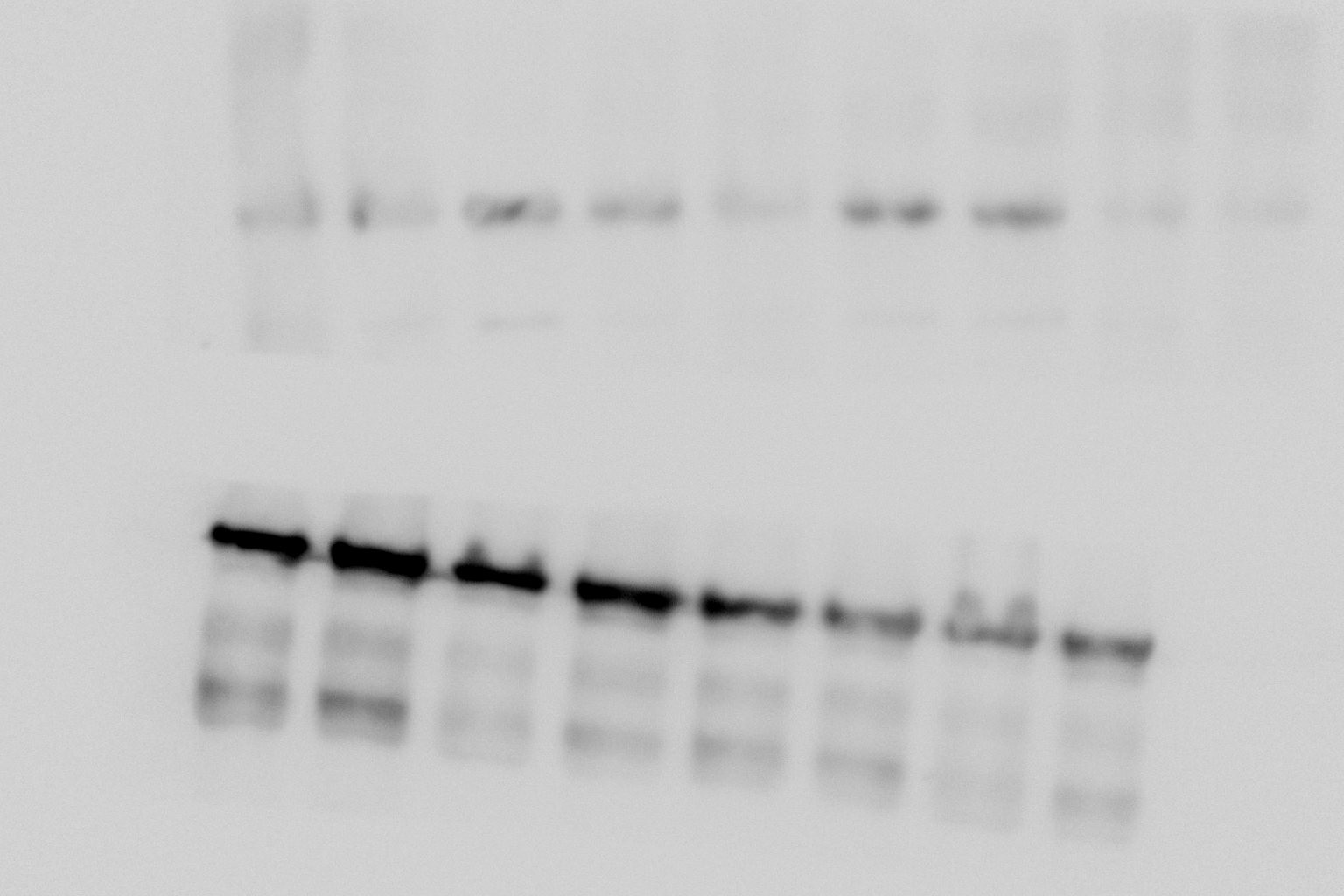


Nuclear HNF4α

Tshr(+/+)

Tshr(-/-)

Tshr(+/+)

Tshr(-/-)


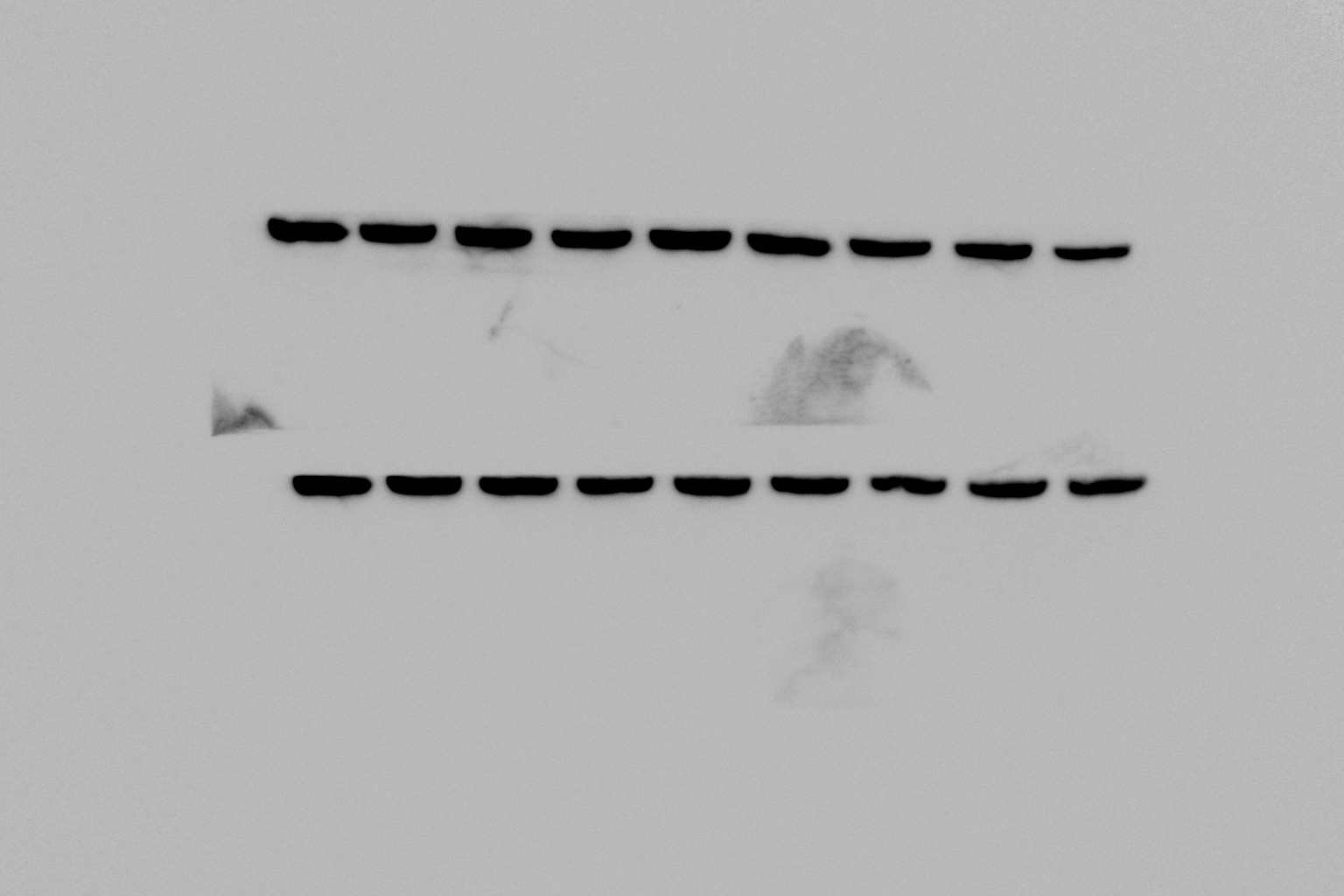


Lamin B 1

Tshr(+/+)

Tshr(-/-)

Tshr(+/+)

Tshr(-/-)
